# Case of Introsusceptio Cured by Forcing Air into the Intestines

**Published:** 1840-07

**Authors:** 


### Selections from American and Foreign Journals.

---

*Case of Introsusceptio cured by forcing Air into the Intestines.*—James Thompson, æt. 44, of a rather spare habit of body, but in the general enjoyment of good health, was suddenly taken ill with pain in his bowels, about ten o'clock in the evening of the 25th Nov. last. He took some spirits, warm drinks, bathed his feet in warm water, and applied warm fomentations to the belly, thinking the pain would wear off. It continued to increase however, and I saw him on the morning of the 29th. I found him laboring under the following symptoms. Pulse full and not particularly hurried—tongue clean—face anxious—belly not distended, and particularly relieved by severe but equitable pressure. No alvine discharge since 4 P. M. the preceding day—pain about the umbilicus most excrutiating—not steady, but at intervals of from three to four minutes, accompanied by severe vomiting and great thirst. I immediately ordered a purgative glyster, which came off almost immediately, bringing with it the contents of the rectum without abating the pain. I ordered its repetition —part returned the same as thrown up, and part remained; but the violent pain still continued. I then applied a blister over the whole surface of the abdomen, and give him a powerful opiate; after an hour this settled the vomiting, and in some degree dulled the pain. I then gave him s. m. hyd. gr. xii. and left him. Twelve hours after the pain and vomiting had returned as violently as ever—still no pain on pressure. I then endeavored to open the bowels by throwing up as much warm water by the domestic machine as I possibly could; he complained of straitness, and part of the water returned without any effect—I then repeated the calomel and left him. On the morning of the 27th, the calomel had proved useless, and the vomiting and pain were as violent as ever; thirst severe, and perspiration profuse. On pressing the belly pain was now felt in the region of the caput cæcum. It was now evident that unless he was speedily relieved death must ensue. I again attempted to overcome the obstruction by throwing up about a gallon of warm water until it was forced back and a considerable part discharged without the least relief. Having some little time ago seen the injection of air suggested in cases similar (I think in a recent Number of the Medico-Chirurgical Review, although I cannot lay my hand upon it now,) I determined to try it. Having nothing at hand but a common bellows, I inserted the tube into the rectum, and commenced gradually to force up the cold air. As soon as it found its way into the intestines, the patient said he felt somewhat easier, and I persevered until I could force no more. In a second or two the rarified air, was forced back with great violence bringing with it the remaining portion of the water I injected, but nothing more. He said he felt rather easier and urged its repetition. This I did for other two different times with no appearance of relief. On the fourth trial, however, the room was filled with a most fetid smell caused by a very free discharge of fæculent matter—he felt relieved, the vomiting ceased, and he complained only of general soreness. I now gave him 8 grs. of cal. with one grain p. opii and left him. That evening he had an alvine discharge, and the following morning he got ol. ricini, ℨj. which operated freely in the course of the day, and the cure was complete.

Whether the foregoing case was real introsusceptio or not I am not prepared to say, but I am sure of this, that, had I not succeeded by the forcing up of air into the intestines, there were no other means that I am aware of whereby I had the least chance to save the poor fellow. I have given the case at this length, as I conceive the plan pursued worthy the attention of the profession in similar cases. It would be absurd to draw a conclusion from a single case, but from what occurred to me in this instance, the practice appears to be perfectly harmless, since the moment the air became rarified it came off with great force, and left no disagreeable consequence behind.—*Medico-Chirurg. Review.*

